# Development of patent *Litomosoides sigmodontis* infections in semi-susceptible C57BL/6 mice in the absence of adaptive immune responses

**DOI:** 10.1186/s13071-015-1011-2

**Published:** 2015-07-25

**Authors:** Laura E. Layland, Jesuthas Ajendra, Manuel Ritter, Anna Wiszniewsky, Achim Hoerauf, Marc P. Hübner

**Affiliations:** Institute of Medical Microbiology, Immunology and Parasitology (IMMIP), University Hospital of Bonn, Sigmund Freud Straße 25, Bonn, 53105 Germany

**Keywords:** *Litomosoides sigmodontis*, Filariae, C57BL/6, Patency, Immune-regulation

## Abstract

**Background:**

One of the most advantageous research aspects of the murine model of filariasis, *Litomosoides sigmodontis*, is the availability of mouse strains with varying susceptibility to the nematode infection. In C57BL/6 mice, *L. sigmodontis* worms are largely eliminated in this strain by day 40 post-infection and never produce their offspring, microfilariae (Mf). This provides a unique opportunity to decipher potential immune pathways that are required by filariae to achieve a successful infection. In this study we tracked worm development and patency, the production of microfilariae and thus the transmission life-stage, in Rag2IL-2Rγ^−/−^ mice which are deficient in T, B and NK cell populations.

**Findings:**

Although worm burden was comparable between wildtype (WT) and Rag2IL-2Rγ^−/−^ mice on d30, by day 72 post-infection, parasites in Rag2IL-2Rγ^−/−^ mice were still in abundance, freely motile and all mice presented high quantities of Mf both at the site of infection, the thoracic cavity (TC), and in peripheral blood. Levels of cytokine (IL-4, IL-6, TNFα) and chemokine (MIP-2, RANTES, Eotaxin) parameters were generally low in the TC of infected Rag2IL-2Rγ^−/−^mice at both time-points. The frequency of neutrophils however was higher in Rag2IL-2Rγ^−/−^mice whereas eosinophils and macrophage populations, including alternatively activated macrophages, were elevated in WT controls.

**Conclusion:**

Our data highlight that adaptive immune responses prevent the development of patent *L. sigmodontis* infections in semi-susceptible C57BL/6 mice and suggest that induction of such responses may offer a strategy to prevent transmission of human filariasis.

**Electronic supplementary material:**

The online version of this article (doi:10.1186/s13071-015-1011-2) contains supplementary material, which is available to authorized users.

## Background

Filariasis remains a major cause of severe morbidity and socioeconomic difficulties in the tropics [1]. Infections persist for numerous years in man due to regulatory strategies developed by the nematode [2]. To understand the principle immune players which govern worm burden and control pathology, researchers use rodent models of the disease such as *Litomosoides sigmodontis* [3]. In BALB/c mice, this model allows the comparison of developing immune responses against all stages of the helminth’s life-cycle, including the release of Mf, the worm’s offspring [4]. C57BL/6 mice on the other hand are refractory to full development of the parasite and progressively eliminate adult worms 40 days post-infection (p.i.) [5, 6]. In all hosts, penetrating *L. sigmodontis* larvae migrate through the lymphatics and settle in the TC after a few days. These larvae then moult into L4 (+9 days p.i.) and then again into adults (+28 days p.i.). During infection, granuloma-like structures encase the filariae but the composition of granulocytes and macrophages within the granulomas does not precisely coincide with the cellular infiltration into the TC [7]. This study followed filarial development in B6-Rag2^tm1Fwa^II2rg^tm1Wjl^ C57BL/6 mice which are deficient in T, B and NK cell populations [8, 9]. *L. sigmodontis* infections in these mice transpired into full patency with long free-living worms and no encapsulation was observed at either d30 or d72 of infection. In addition, whereas the frequencies of eosinophils and RELMα-positive AAM (alternatively activated macrophage) populations were elevated in WT mice in the TC, neutrophil populations were most prominent in Rag2IL-2Rγ^−/−^ C57BL/6 mice. Although previous studies have indicated that parasite clearance in non-susceptible mice is Th2 dependent [10], our new findings indicate that other immune factors are also required such as NK cell populations.

## Findings

### Methods

#### Animals, ethics and infection

B6-Rag2^tm1Fwa^II2rg^tm1Wjl^ mice (http://www.taconic.com/4111) were purchased from Taconic Biosciences Inc, Cologne, Germany and bred alongside WT C57BL/6 mice. Mice were kept under SPF conditions and experiments were in accordance with local government authorities (No.87-51.04.2011.A025/01). Infections and parasite recovery with *L. sigmodontis* were performed as previously described [11]. Levels of *Wolbachia* DNA were performed using a duplex PCR measuring *Wolbachia* FtsZ and Actin [12].

#### Flow cytometry staining of TC cells

Isolated TC cells were prepared for flow cytometry as previously described [11]. In short, fixed cells were stained with SiglecF-PE (BD Bioscience, Heidelberg, Germany), F4/80-PerCP-Cy5.5, Gr1-PE-Cy7, CD86-FITC and MHC II-APC (all eBioscience, San Diego, USA). AAM were identified using intracellular staining for RELMα with rabbit anti-mouse RELMα (Peprotech, Rocky Hill, USA) followed by goat anti-rabbit Alexa Fluor 488-conjugated antibody (Invitrogen, Carlsbad, USA). Gating strategies are shown in Additional file 1. Flow cytometry was performed using a BD FACS Canto I and analyzed using FACSDiva 5.2 software (BD Biosciences).

#### Cytokine determination

Cytokine concentrations in the TC fluid were measured using ELISA in accordance to the manufacturer’s instructions (IL-6, RANTES, TNF-α, MIP-2 and Eotaxin all from RnD Systems, Minneapolis USA; IL-4 from BD Biosciences). ELISA plates were read and analyzed at 450 and 540 nm (Molecular Devices, Sunnyvale, CA, USA).

#### Statistical analysis

Statistical differences were determined using GraphPad Prism 5 software (San Diego, CA, USA). Parametrically distributed data were analyzed using unpaired t-tests or one-way ANOVA whereas non-parametrically distributed data were calculated using Kruskal-Wallis-tests. If significant, this was followed by a Mann–Whitney–U test for a further comparison of the groups.

## Results & discussion

### Patent *L. sigmodontis* infection in Rag2IL-2Rγ-deficient C57BL/6 mice

**Fig. 1:**
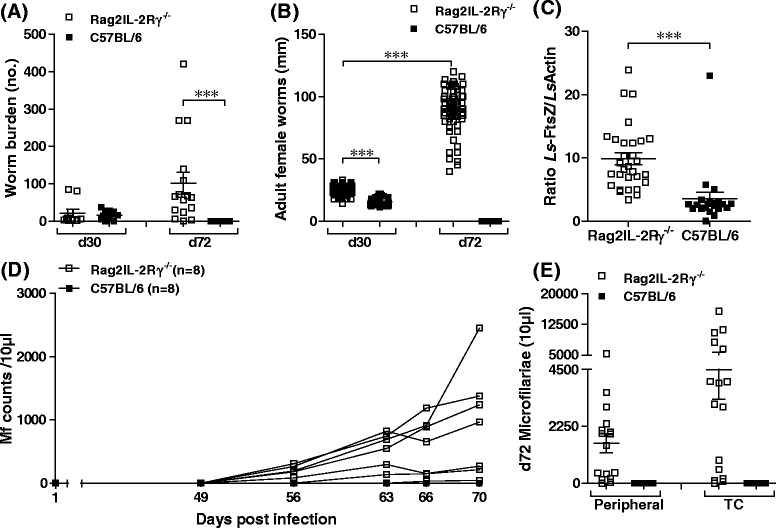
*L. sigmodontis* infection in RAG2IL-2Rγ^−/−^ C57BL/6 mice leads to patency. Groups of male WT and Rag2IL-2Rγ^−/−^ C57BL/6 mice were naturally infected with *L. sigmodontis*. After d30 and 72 p.i. mice were assessed for absolute worm burden (**a**) and adult female worm length (**b**). Levels of *Wolbachia* DNA were determined via a duplex PCR in individual adult female worms (n = 30 Rag2IL-2Rγ^−/−^ and n = 21 C57BL/6) from d30 p.i. (**c**). Peripheral levels of microfilariae were detected between d49-70 p.i. (**d**) and on d72 p.i. in blood and TC fluid (**e**). Symbols in B and C show values from individual worms. Data in A shows values from individual mice on day 30 from n = 10 WT and n = 10 Rag2IL-2Rγ^−/−^ (one infection study) and on d72 in n = 20 WT and n = 16 Rag2IL-2Rγ^−/−^ (three independent infection experiments). Data in D shows Mf counts in n = 8 WT and n = 8 Rag2IL-2Rγ^−/−^ mice within the same infection experiment. E shows Mf counts in individual mice on d72 (n = 20 WT and n = 16 Rag2IL-2Rγ^−/−^) from three independent infection experiments. Asterisks denote significant differences between the groups indicated by the brackets (***p < 0.001)

Male Rag2IL-2Rγ^−/−^ and WT C57BL/6 mice were naturally infected with *L. sigmodontis*. After 30 days, mice were assessed for worm burden (Fig. 1a) and stage (Additional file 2A) since this time-point corresponds with the final moulting phase from L4 into adulthood. Although no significant differences in total worm burden were observed on d30 (Fig. 1a), Rag2IL-2Rγ^−/−^ mice had significantly higher numbers of adult worms compared to L4 (Additional file 2A) indicating an earlier moult into adulthood. On day 72, worms in all WT mice had been encapsulated into granulomatous nodules that were absence in Rag2IL-2Rγ^−/−^ mice. Filariae in Rag2IL-2Rγ^−/−^ mice however were freely motile and in abundance (Fig. 1a). Both adult worm genders in Rag2IL-2Rγ^−/−^ mice were also significantly longer in length and substantially grew between d30 and 72 (Fig. 1b and Additional file 2B). Levels of *Wolbachia* DNA in adult female worms were also significantly higher than in females from control mice (Fig. 1c). Their healthy state was further reflected in the production of Mf (Fig. 1d and Additional file 2C). Indeed, from day 49 p.i., increasing numbers of Mf could be detected in the periphery of all infected Rag2IL-2Rγ^−/−^ mice (n = 16 in three independent infection experiments) whereas none occurred in WT mice (Fig. 1d and Additional file 2C). Fig. 1e also shows correspondingly high levels of Mf in the TC of KO mice on the day of analysis (d72) and Mf length was 91.2 ± 6.1 μm. Previous studies have demonstrated the development of *L. sigmodontis* patency in IL-4^−/−^ C57BL/6 mice. One study demonstrated that a s.c. injection of L3 into IL-4-deficient C57BL/6 mice resulted in a patent state in 33 % of *L. sigmodontis*–infected mice [10]. Further studies have shown that IL-10 counter-regulates these effects since IL-4/IL-10^−/−^ mice failed to develop a patent state [13]. Another study using RAG2/IL-4-deficient mice reported significantly higher microfilariae levels than in susceptible BALB/c mice, although the prevalence of patency was not discussed [14]. Our study highlights therefore, that lack of T, B and NK cells suffices to render C57BL/6 mice susceptible, leading to a 100 % prevalence of patent animals that exceeds the impact of a single IL-4-deficient mice. Since *L. sigmodontis*-infected μMT C57BL/6 mice do not develop patent infections it appears that a lack of B cells does not facilitate the development of patency [10]. Nevertheless, the importance of B and T cells was previously demonstrated in infections with *L. sigmodontis* susceptible BALB/c mice, since B1 cell-deficient (Xid) and CD4^+^ T cell depleted mice had increased worm burden and microfilariae [15, 16]. However, lack of IL-4 in BALB/c mice also increased patency but not worm load [17] indicating that IL-4-mediated immune responses hinder Mf release but not necessarily worm development.

### Elevated levels of MIP-2 in the TC of C57BL/6 mice upon elimination of *L. sigmodontis* filariae

**Fig. 2:**
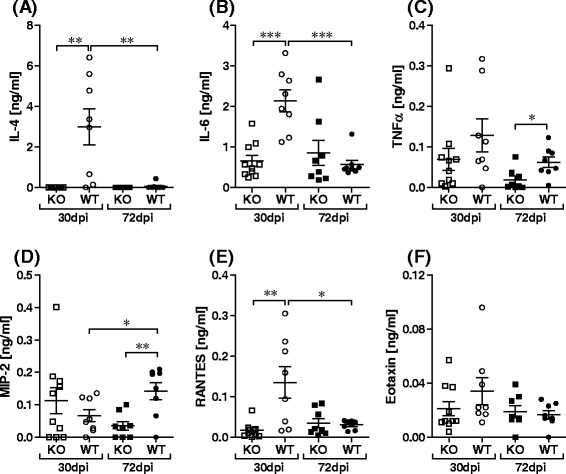
Low levels of cytokine and chemokines in the TC of *L. sigmodontis*-infected Rag2IL-2Rγ^−/−^ C57BL/6 mice. Levels of IL-4 (**a**), IL-6 (**b**), TNF-α (**c**), MIP-2 (**d**), RANTES (**e**) and Eotaxin-1 (**f**) were measured in the TC fluid from individual mice on days 30 and 72 p.i. by ELISA. Symbols show levels in individual mice. Graphs show data from one infection experiment comprising of n = 10 WT and n = 10 Rag2IL-2Rγ^−/−^ on d30 and n = 8 WT and n = 8 Rag2IL-2Rγ^−/−^ on d72. Asterisks denote significant differences n = 10 between the groups indicated by the brackets (*p < 0.01, **p < 0.05, ***p < 0.001)

As IL-4 is critical for sexual maturity and Mf release in C57BL/6 mice [10, 13], we determined IL-4 levels in the TC on d30 and 72 in infected groups. Whereas no IL-4 could be detected in Rag2IL-2Rγ^−/−^ mice at either time-point, levels in WT mice were significantly elevated on d30 when compared to the Rag2IL-2Rγ^−/−^ group and WT mice on d72 (Fig. 2a). Based on the differences in frequency of patent animals between IL-4 deficient and Rag2IL-2Rγ^−/−^ mice, we assume that the lack of T and B cells exceeds the importance of IL-4 alone in Rag2IL-2Rγ^−/−^ mice accounting for the observed phenotype. Interestingly levels of another Th2 cytokine, IL-13, were previously shown to be comparable in *L. sigmodontis-*infected WT and IL-4^−/−^ mice [13]. In the study here, levels of IL-6 in the TC of WT mice were also significantly higher on d30 than d72 (Fig. 2b) but levels of TNF-α in WT mice were only significantly elevated on d72 p.i. when compared to Rag2IL-2Rγ^−/−^ mice (Fig. 2c). This pattern was also observed with levels of MIP-2 and interestingly, levels of this chemokine were also significantly elevated in WT mice on d72 when compared to WT mice on d30 (Fig. 2d). Levels of RANTES in the TC, reflected measurements of IL-4, that is, significantly higher amounts on day 30 when compared to d72 (Fig. 2e). Levels of Eotaxin-1 however were very low in all groups at both time-points (Fig. 2f). TC levels of these parameters were analysed in two further independent infection experiments on d72 (n = 10 WT and n = 8 Rag2IL-2Rγ^−/−^ mice) and levels were comparable to those depicted in Fig. 2 (data not shown). Those results are in accordance to the well described role of T and B cells to drive immune responses.

### *L. sigmodontis*-infected Rag2IL-2Rγ^−/−^ C57BL/6 mice present elevated neutrophils but reduced AAM populations at the site of infection

**Fig. 3:**
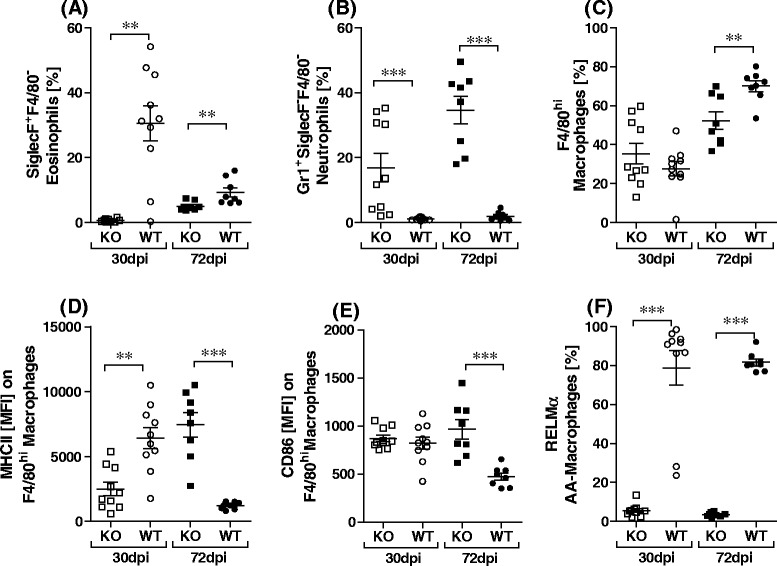
Elevated neutrophils but reduced AAM populations in *L. sigmodontis*-infected Rag2IL-2Rγ^−/−^ C57BL/6 mice. TC cells, isolated from individual mice on d30 or 72p.i. were assessed for the frequency of SiglecF^+^F4/80^−^ eosinophils (**a**), Gr1^+^SiglecF^−^F4/80^−^neutrophils (**b**) and F4/80^+^ macrophages (**c**). Macrophage populations were then further subdivided into F4/80^hi^MHCII^+^ (**d**), F4/80^hi^CD86^+^ (**e**) and RELM-α^+^ (**f**). Symbols show frequency (**a-c, f**) or MFI (**d, e**) levels in individual mice at both time-points (10 WT and n = 10 Rag2IL-2Rγ^−/−^ on d30 and n = 8 WT and n = 8 Rag2IL-2Rγ^−/−^ on d72) from one infection study. Asterisks denote significant differences n = 10 between the groups indicated by the brackets (**p < 0.05, ***p < 0.001)

Next we studied changes in immune cell populations in the TC. At both analysed time-points, SiglecF^+^F4/80^−^ eosinophil populations remained significantly elevated in WT mice (Fig. 3a), whereas Gr1^+^SiglecF^−^F4/80^−^ neutrophils were higher in Rag2IL-2Rγ^−/−^ mice (Fig. 3b). The role of neutrophils and eosinophils in controlling filarial development and granuloma development has been well studied [18–20]. The elevated frequency of neutrophils in Rag2IL-2Rγ^−/−^ mice here suggests that neutrophils require further signals to develop granulomas and/or control worm burden. Since Rag2IL-2Rγ^−/−^ mice also lack NK populations it will be interesting to observe the effects on worm burden in these mice upon reconstitution of IL-4 competent NK cells. The actual role of NK cells in filarial infections is not well-defined although studies with *L. sigmodontis*, have shown that depletion of NK cells enhanced worm load and Th2 responses at the site of infection [21]. This correlates to the findings observed here with Rag2IL-2Rγ^−/−^ mice since they also lack NK cell populations. With regards to macrophage populations, the percentage of F4/80^hi^ cells was significantly higher in WT mice on d72 of infection (Fig. 3c). However, MHCII and CD86 expressing F4/80^hi^ populations were elevated in Rag2IL-2Rγ^−/−^ mice on d72 p.i., highlighting an increased activation (Figs. 3d and e respectively). Finally, we also determined the frequency of AAM population using RELM-α, at both time-points, this population was significantly higher in WT mice (Fig. 3f). Previous studies have elucidated that in the absence of recruitment signalling, IL-4 drives macrophage proliferation and reduces the development of AAM populations [22]. Interestingly, acute infected *L. sigmodontis* Rag1^−/−^ C57BL/6 mice showed no development of Ym1^+^ (AAM) [22] which correlates to the lack of AAM in the Rag2IL-2Rγ^−/−^ mice studied here, demonstrating that adaptive immune responses appear fundamental for AAM development. Flow cytometry data in two further independent infection experiments analysed on d72 (n = 10 WT and n = 8 Rag2IL-2Rγ^−/−^ mice) were comparable (data not shown). Future studies will have to analyze the impact of specific cell populations including AAM, T cell subsets, B cells and NK cells using Rag-deficient strains to further elucidate mechanisms that could be targeted to prevent the development of patent infections.

## Conclusion

In the current study we demonstrate that deficiency in T, B, and NK cells renders semi-susceptible C57BL/6 mice to 100 % patent during *L. sigmodontis* infection. Using the *L. sigmodontis* Rag2IL-2Rγ^−/−^ model offers therefore the opportunity to identify cell types that are involved in the development of patency, presenting new targets to combat filarial transmission.

## Additional files

Additional file 1:
**Gating strategies for flow cytometry.** Groups of male WT and Rag2IL-2Rγ^−/−^ C57BL/6 mice were infected with *L. sigmodontis*. After 72 days of infection, cells from the TC were isolated and screened for different cell populations by flow cytometry. A-C: Gating Strategy for TC cells, gates for macrophages, eosinophils, neutrophils. D: Gating strategy for AAM and E: Plots for expression of CD86 and MHCII in macrophages. (PPT 142 kb) Additional file 2:
**Infections with** ***L. sigmodontis*** **in Rag2IL-2Rγ** ^**−/−**^ **C57BL/6 mice drives faster moulting into adulthood.** Groups of male WT and Rag2IL-2Rγ^−/−^ C57BL/6 mice were infected with *L. sigmodontis*. After d30 p.i. worms were assessed for larval or adult life-stages **(A)**. Symbols show the number of different worm stages recovered from individual mice (n = 10 WT and n = 10 Rag2IL-2Rγ^−/−^). **(B)**, on d30 and d72, adult male worm length was determined. **(C)** Peripheral levels of microfilariae were determined from d49 until d70 p.i. Each symbol represents the Mf load in individual mice (n = 10 WT and n = 8 Rag2IL-2Rγ^−/−^) from two independent infection experiments. Asterisks denote significant differences between the groups indicated by the brackets (***p < 0.001). (PPTX 63 kb)

## Abbreviations

*L. sigmodontis*: *Litomosoides sigmodontis*
Mf: Microfilariae
TC: Thoracic cavity
