# Supplementary figures and images for: Development of patent Litomosoides sigmodontis infections in semi-susceptible C57BL/6 mice in the absence of adaptive immune responses
Source: Parasit Vectors. 2015 Jul 25;8:396. doi: 10.1186/s13071-015-1011-2 (PMC4514938; doi:10.1186/s13071-015-1011-2)

## Slide 1
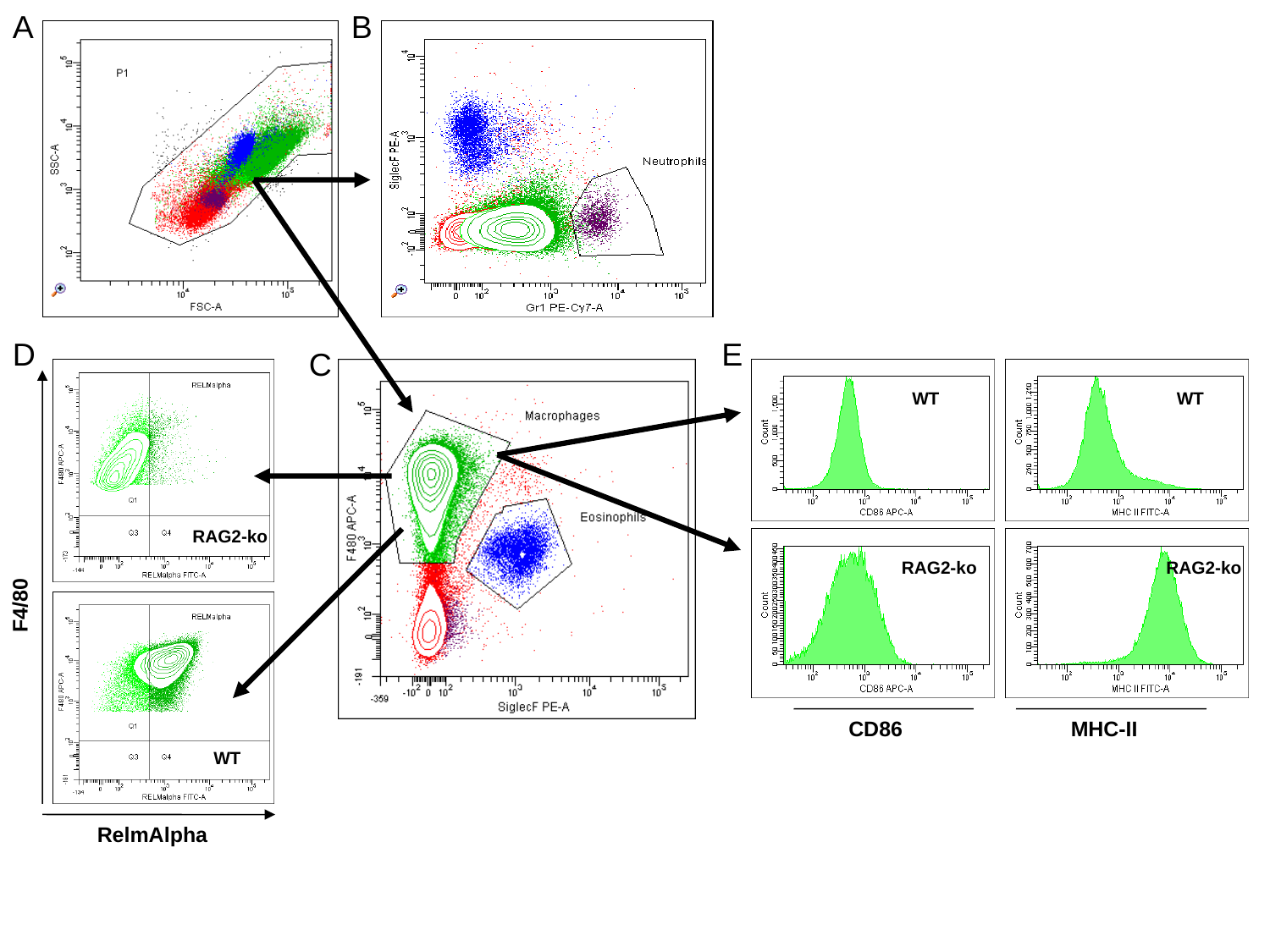

A
B
D
E
C
WT
WT
F4/80
RAG2-ko
RAG2-ko
RAG2-ko
CD86
MHC-II
WT
RelmAlpha

Supplement: Additional file 1: — Gating strategies for flow cytometry. Groups of male WT and Rag2IL-2Rγ−/− C57BL/6 mice were infected with L. sigmodontis. After 72 days of infection, cells from the TC were isolated and screened for different cell populations by flow cytometry. A-C: Gating Strategy for TC cells, gates for macrophages, eosinophils, neutrophils. D: Gating strategy for AAM and E: Plots for expression of CD86 and MHCII in macrophages. (PPT 142 kb) [file 13071_2015_1011_MOESM1_ESM.ppt]

## Slide 1
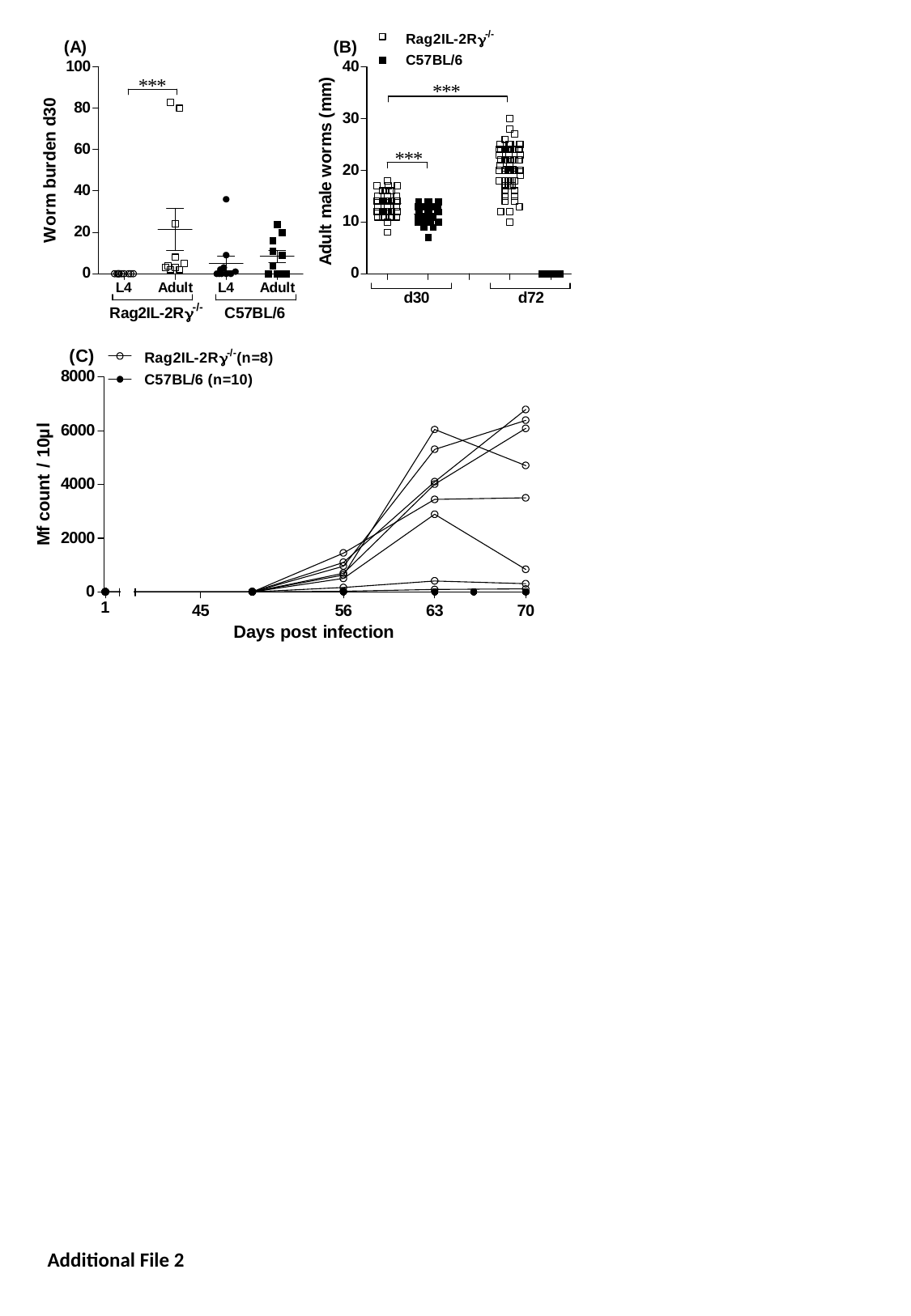

Additional File 2

Supplement: Additional file 2: — Infections with L. sigmodontis in Rag2IL-2Rγ −/− C57BL/6 mice drives faster moulting into adulthood. Groups of male WT and Rag2IL-2Rγ−/− C57BL/6 mice were infected with L. sigmodontis. After d30 p.i. worms were assessed for larval or adult life-stages (A). Symbols show the number of different worm stages recovered from individual mice (n = 10 WT and n = 10 Rag2IL-2Rγ−/−). (B), on d30 and d72, adult male worm length was determined. (C) Peripheral levels of microfilariae were determined from d49 until d70 p.i. Each symbol represents the Mf load in individual mice (n = 10 WT and n = 8 Rag2IL-2Rγ−/−) from two independent infection experiments. Asterisks denote significant differences between the groups indicated by the brackets (***p < 0.001). (PPTX 63 kb) [file 13071_2015_1011_MOESM2_ESM.pptx]
